# Genetic Variability of Bovine Viral Diarrhea Virus and Evidence for a Possible Genetic Bottleneck during Vertical Transmission in Persistently Infected Cattle

**DOI:** 10.1371/journal.pone.0131972

**Published:** 2015-07-01

**Authors:** Natalie Dow, Adam Chernick, Karin Orsel, Guido van Marle, Frank van der Meer

**Affiliations:** 1 Department of Ecosystem and Public Health, Faculty of Veterinary Medicine, University of Calgary, Calgary, Alberta, Canada; 2 Department of Production Animal Health, Faculty of Veterinary Medicine, University of Calgary, Calgary, Alberta, Canada; 3 Department of Microbiology, Immunology and Infectious Disease, Cumming School of Medicine, University of Calgary, Calgary, Alberta, Canada; 4 Department of Community Health Sciences, Cumming School of Medicine, University of Calgary, Calgary, Alberta, Canada; Texas A&M Veterinary Medical DIagnostic Laboratory, UNITED STATES

## Abstract

Bovine viral diarrhea virus (BVDV), a *Pestivirus* in the family Flaviviridae, is an economically important pathogen of cattle worldwide. The primary propagators of the virus are immunotolerant persistently infected (PI) cattle, which shed large quantities of virus throughout life. Despite the absence of an acquired immunity against BVDV in these PI cattle there are strong indications of viral variability that are of clinical and epidemiological importance. In this study the variability of E2 and NS5B sequences in multiple body compartments of PI cattle were characterized using clonal sequencing. Phylogenetic analyses revealed that BVDV exists as a quasispecies within PI cattle. Viral variants were clustered by tissue compartment significantly more often than expected by chance alone with the central nervous system appearing to be a particularly important viral reservoir. We also found strong indications for a genetic bottleneck during vertical transmission from PI animals to their offspring. These quasispecies analyses within PI cattle exemplify the role of the PI host in viral propagation and highlight the complex dynamics of BVDV pathogenesis, transmission and evolution.

## Introduction

Bovine viral diarrhea virus (BVDV) is a major production limiting disease of cattle due to the clinical signs following infection [1] and the associated economic consequences [2]. BVDV is a *Pestivirus* belonging to the family Flaviviridae and can be divided into two genotypes: BVDV 1 and 2. Subgenotypes BVDV 1a, 1b and 2a have been identified as the dominant circulating strains in North America [3, 4]. Independent of genotype, BVDV can also be further classified as one of two biotypes, cytopathic (CP) or noncytopathic (NCP), based on the ability of the virus to lyse cells in tissue culture [5]. The NCP BVDV biotypes are of particular clinical importance, as they are capable of crossing the placenta of an acutely infected dam. Transplacental infection of the fetus between approximately 30 and 120 days of age can lead to persistent infection (PI) of the calf [6, 7]. While both transiently and persistently infected dams can produce PI calves, every calf produced by a PI dam will be persistently infected [8]. Due to viral establishment before maturation of the fetal immune system, PI calves will remain immunotolerant of the BVDV strain as the viral proteins are regarded as self-antigens, allowing for viral replication in all tissues and excretions without host detection [9]. High levels of viral shedding [10] further emphasizes the importance of PI cattle as the most significant propagators of BVDV. Despite this, very little is known regarding the viral population structure in these animals. Persistent infections of viruses such as human immunodeficiency virus (HIV, a *Lentivirus*) or Hepatitis C virus (HCV, a *Hepacivirus*) in an immunocompetent host rely on various mechanisms to continuously escape neutralization by host antibodies [11–13]. Within the PI host, BVDV replicates without the selective influence of the adaptive immune system, therefore PI animals represent a unique evolutionary model.

One possible outcome of efficient adaptation to the host is compartmentalization of viral variants as demonstrated by HIV [14, 15], where viral populations are genetically distinct between different body compartments due to local selection pressures. Compartmentalization has also been demonstrated in HCV [16, 17], another member of the *Flavivirus* genus. Most HCV compartmentalization studies focus on the hypervariable region 1 (HVR1) located in the N-terminal region of the E2 glycoprotein, which showed that many patients harbour viral variants with distinct cellular tropisms between populations in the liver, serum, and peripheral blood mononuclear cells (PBMCs).

Analysis of intrahost virus variability provides insight into localized evolutionary drivers and can reveal important determinants of viral pathogenesis that contribute to disease outcome. This virus population diversity is considered important in RNA virus evolution and survival and is attributed to the error prone genome replication by the RNA-dependent RNA polymerase (RdRP) [18]. Previous studies have indicated that intrahost viral variability occurs in BVDV PI cattle. Early in experimentally infected PI cattle an increase of variability over time was demonstrated in the E2 membrane glycoprotein [19]. Low frequency variants in the 5’UTR were also detected in different tissues of a PI fetus [20] and, more recently, one study showed that genetic diversity increased more rapidly in PI animals than in multiple transient BVDV infections [21].

Our study focused on the N-terminal region of the ectodomain of the variable E2 [19] structural protein and the non-structural RdRP protein encoded by the relatively conserved NS5B [22]. The N-terminal E2 region harbours dominant neutralizing epitopes and plays a vital role in cell fusion and binding [23]. The goal of this study was to describe the extent and distribution of BVDV variability in multiple body compartments of naturally infected PI cattle derived from Western Canadian dairy herds. Moreover, we describe the quasispecies diversity in the progeny of PI cattle, suggesting a genetic bottleneck following vertical transmission from PI dam to fetus.

## Results

### Cloning and Sequencing

A total of 10 PI cattle were identified across five Western Canadian dairy herds (Table 1). A minimum of 10 E2 and NS5B sequences were obtained from colon, ileum, milk, mesenteric lymphnode (MLN), obex, and tonsil tissues as well as serum. NS5B clones from the colon, ileum, and obex of PI6 and PI7 could not be obtained as they were not positive by PCR amplification. All sequences are available in GenBank (accession numbers KP755034-KP756413). The phylogenetic analysis of E2 consensus sequences (S1 Fig) showed that viruses from different farms clearly segregated into different clusters, while PI cattle from the same farm produced identical consensus sequences consistent with previous work suggesting that PI cattle establish herd specific BVDV strains [24, 25]. However, as changes in virus populations are not necessarily reflected in changes to the consensus sequence [26], the use of clonal analysis was justified to further study the intrahost construct.

**Table 1:** Summary of all cattle included in the study as well as the data generated from clonal sequencing.

PI ID	Farm ID	Family ID	BVDV Subgenotype	No. Of E2 Clones	No. Of NS5B Clones
1	1	NA	1a	76	75
2	1	F1-G1	1a	78	79
3	1	F1-G2	1a	75	81
4*	1	F1-G3	1a	72	70
5	2	NA	1b	69	63
6	3	NA	1b	67	31
7	3	NA	1b	70	30
8	4	NA	1a	72	69
9	5	F2-G1	1b	80	82
10**	5	F2-G2	1b	73	68

* Fetus in approximately the fourth month of gestation

** Fetus in approximately the eighth month of gestation

NA: not applicable, not part of a family

**Fig 1:**
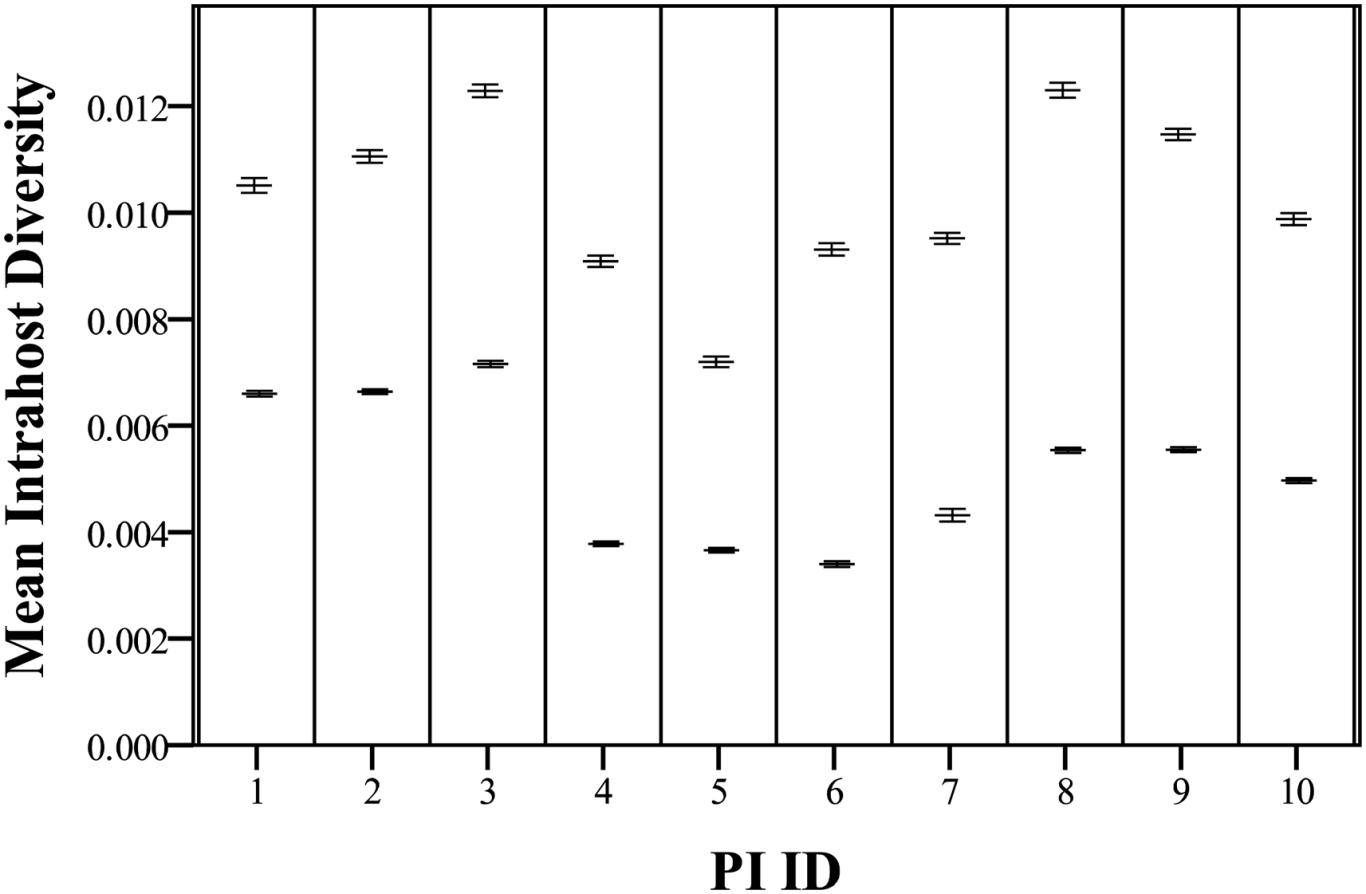
Mean total intrahost E2 (upper bar) and NS5B (lower bar) distances for each PI animal. Distance are based on a minimum of 70 sequences per gene per animal. Horizontal bars indicate the mean distances and the error bars represent the standard error of the mean (SEM). See S2 Table for the exact values.

Unique PI ID numbers were assigned to animals as well as an additional designation (Family ID) for those animals that were part of a family within this sample. F denotes the family and G denotes the generation. For example, F1-G2 is the second generation of family 1. Furthermore, PI4 is the fetus of PI3 and PI10 is the fetus of PI9. The BVDV genotype was determined by comparison of the consensus E2 sequence to the reference sequences depicted in Fig 1.

### Phylogenetic Analysis

Average diversities (as represented by the average pairwise distance between aligned sequences) for both gene fragments were calculated at the host level (Fig 1) and at the tissue level for each host. Both gene regions exhibited similar patterns of change when comparing between individual animals. However, E2 sequence alignments of all compartments in a single host are an average of 1.98 times more diverse than NS5B sequence alignments; E2 is significantly more diverse than NS5B when compared with a paired t-test (t(9) = 16.6, p<0.0001). Within PI family 1, PI4 (fetus, F1-G3) shows significantly less diversity than both its mother (PI3/F1-G2) and grandmother (PI2/F1-G1) for E2 alignments according to a one-way ANOVA (F(2) = 193.9, p<0.0001) and Tukey’s HSD post-hoc testing (all pairwise comparisons were significant at p<0.0001) as well as NS5B alignments (F(2) = 1200.2, p<0.0001 with all pairwise comparisons significant at p<0.0001). A significant decrease is also seen when comparing PI10 (fetus, F2-G2) to PI9 (mother, F2-G1) with paired t-tests for E2 (t(151) = 10.0, p<0.0001) and NS5B (t(148) = 9.0, p<0.0001) alignments, although the difference is less pronounced. In addition to differences in diversity it was also noted that F2-G2 gave rise to six novel mutations that were not observed in the progenitor PI F2-G1, four of which were non-synonymous (S1 Table). Analysis of individual tissue compartments across all PI animals showed that diversity was not different based on NS5B alignments using a one-way ANOVA (F(6) = 1.2, p = 0.3415) but was based on E2 alignments (F(6) = 2.9, p = 0.0163). Tukey’s HSD results are presented in Table 2. Of note are the obex-derived sequences which tend to be more diverse than other compartments.

**Table 2:** Tukey’s HSD post-hoc testing for differences in diversity between different tissue compartments across all PI animals using the E2 alignment.

Group 1	Group 2	Difference	p-value
Colon	Ileum	0.0014	0.9177
Colon	Milk	0.0026	0.7735
Colon	MLN	0.0010	0.9836
Colon	Obex	0.0044	0.0147*
Colon	Serum	0.0005	0.9996
Colon	Tonsil	0.0003	>0.9999
Ileum	Milk	0.0012	0.9945
Ileum	MLN	-0.0004	0.9999
Ileum	Obex	0.0030	0.2168
Ileum	Serum	-0.0009	0.9905
Ileum	Tonsil	-0.0011	0.9735
Milk	MLN	-0.0016	0.9740
Milk	Obex	0.0018	0.9393
Milk	Serum	-0.0021	0.9043
Milk	Tonsil	-0.0023	0.8590
MLN	Obex	0.0034	0.1120
MLN	Serum	-0.0005	0.9996
MLN	Tonsil	-0.0007	0.9976
Obex	Serum	-0.0039	0.0431*
Obex	Tonsil	-0.0041	0.0284*
Serum	Tonsil	-0.0002	>0.9999

* p<0.05

Pairwise comparisons of between all tissues were performed following a significant one-way ANOVA analysis. Mean diversities were calculated for each compartment by taking the mean of the diversities for that compartment in each PI animal. Each animal was weighted equally regardless of the number of clones available for that individual.

**Fig 2:**
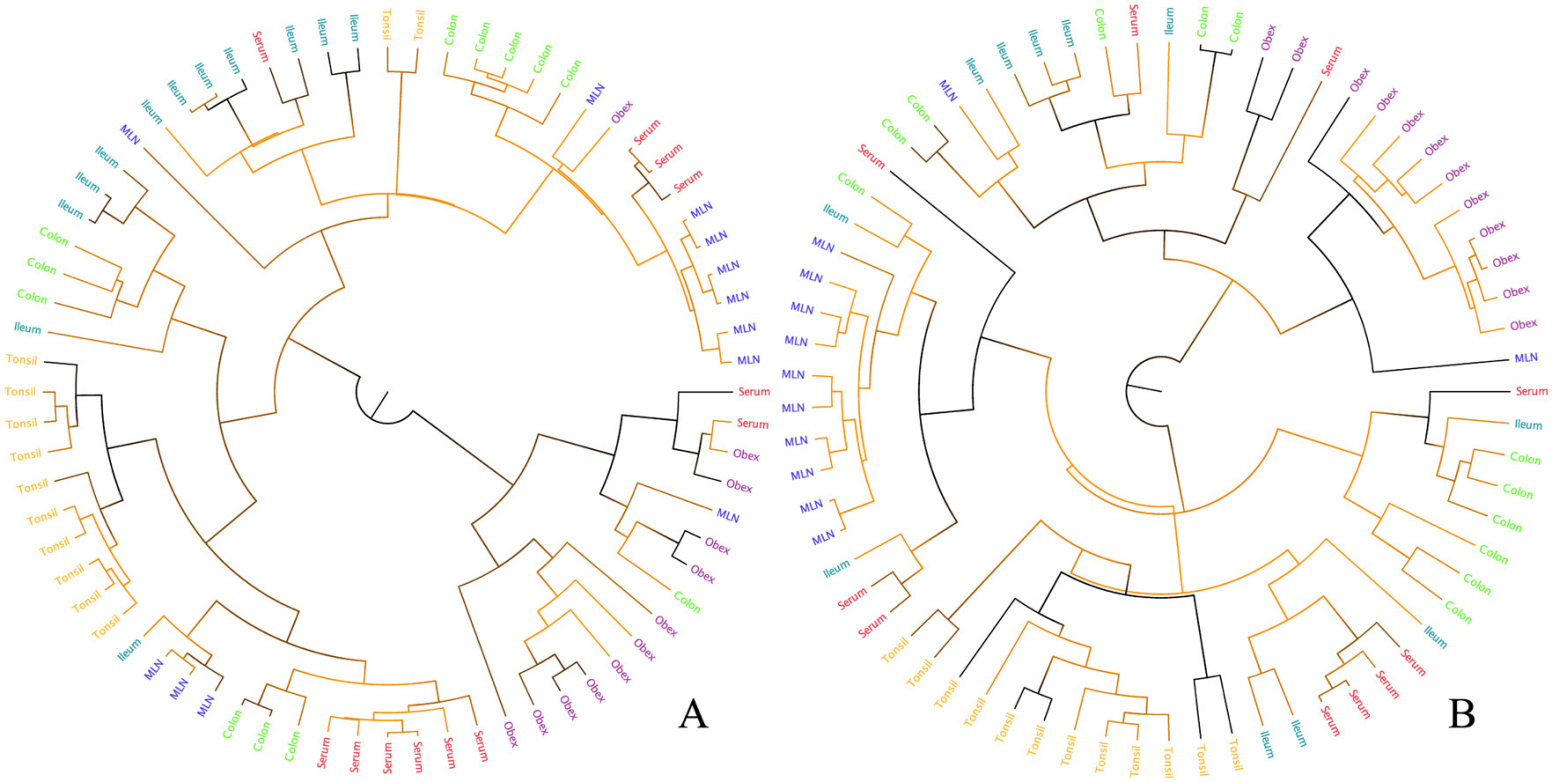
Example phylogenetic tree based on E2 (A) and NS5B (B) gene fragment alignment for PI8. See S2 (E2) and S3 (NS5B) Figs for all other trees. Branches are shaded according to their posterior probability (0 = orange, 1 = black) and the tip labels are coloured according to their tissue of origin (tonsil = yellow, colon = green, MLN = blue, obex = purple, serum = orange, ileum = teal).

Phylogenies were inferred for all animals sampled for both the gene fragments (Fig 2, S2 and S3 Figs). There is strong clustering of taxa by their tissue of origin as discussed below. However, there is generally low support for most internal branches and some recent ones which limits the conclusions that can be made based on these trees.

**Fig 3:**
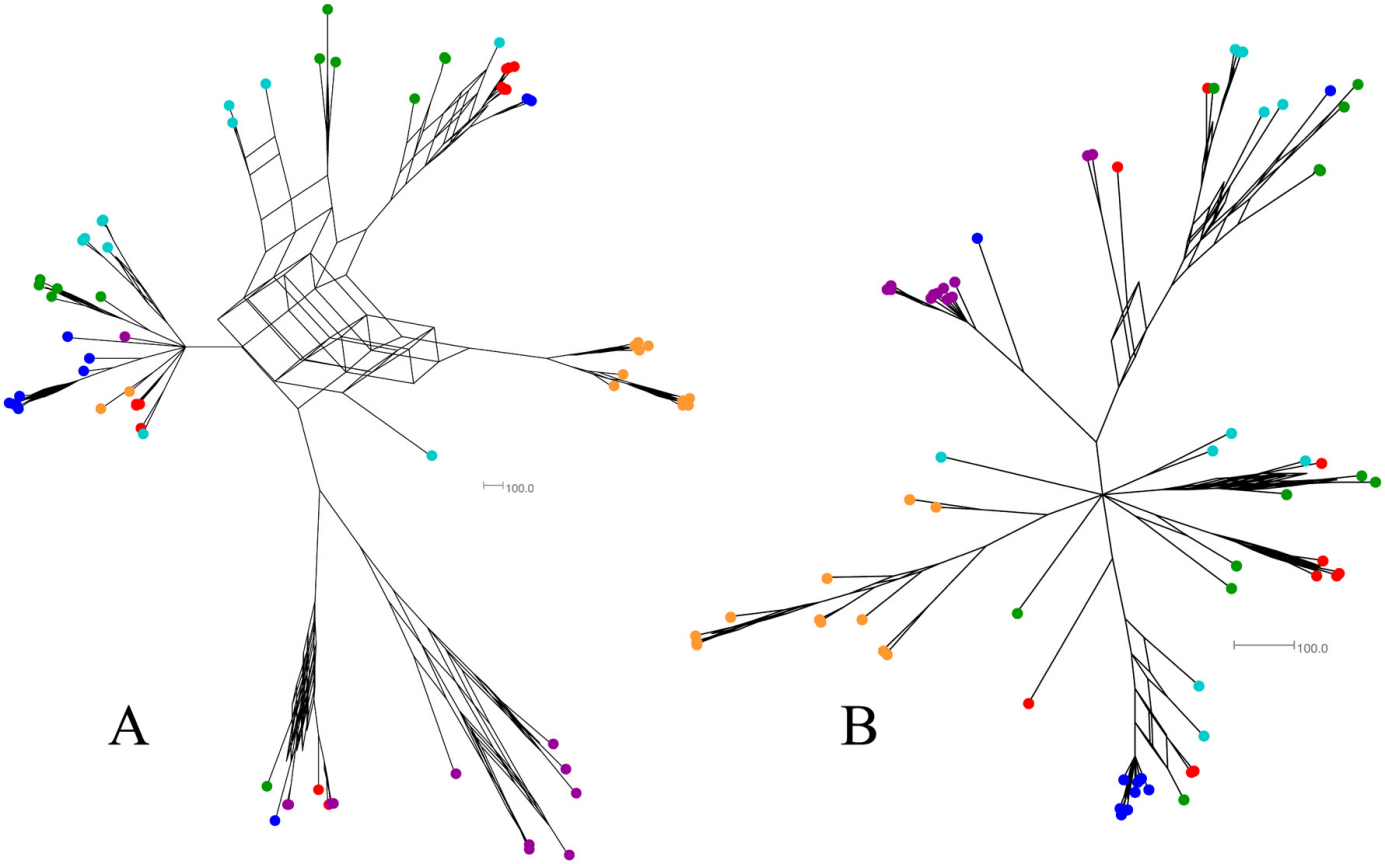
Example consensus network based on the E2 (A) and NS5B (B) gene fragment posterior tree sets for PI8. Tips are coloured according to their tissue of origin (tonsil = yellow, colon = green, MLN = blue, obex = purple, serum = orange, ileum = teal).

Consensus networks largely agree with the more recent branching patterns seen in consensus trees but differ in the more ancestral events (Fig 3, S4 and S5 Figs). The networks display a star-like pattern of evolution which is consistent with the introduction of a small, homogenous population of viral particles that rapidly spread to infect different body compartments prior to any significant genetic drift or mutation. Although the support for the splits in these networks (not shown for clarity) is not necessarily higher than the support shown on the trees, the networks are able to display more potential evolutionary relationships rather than just one. This makes them more suitable for evaluating instances like this where there are multiple, reasonable evolutionary events detected using Bayesian phylogenetics.

### Analysis of PI Families

**Fig 4:**
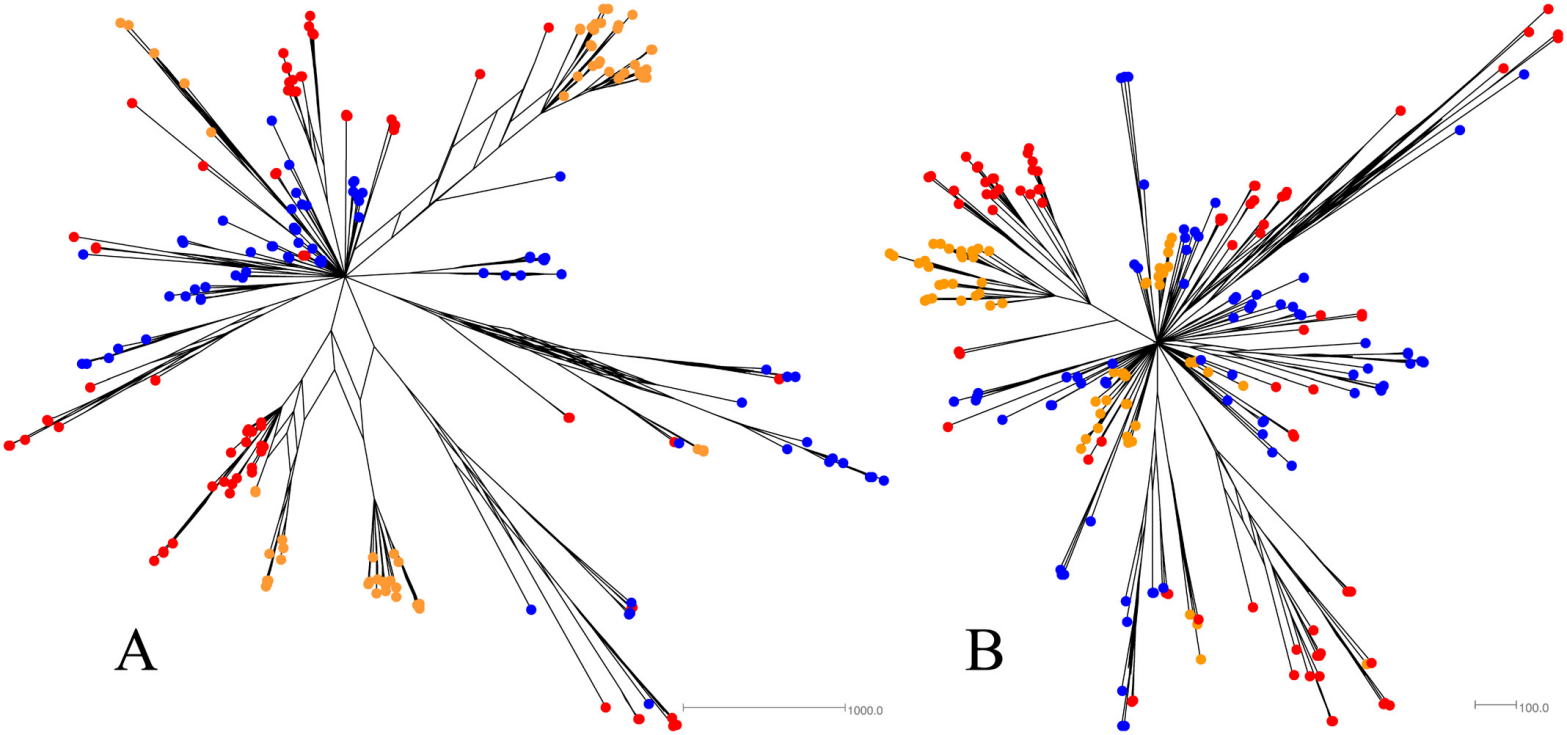
Consensus networks based on the posterior sets of trees for PI family 1. E2 (A) and NS5B (B) sequence alignments were evaluated independently. F1-G1 tips are orange, F1-G2 tips are blue, and F1-G3 tips are red.

**Fig 5:**
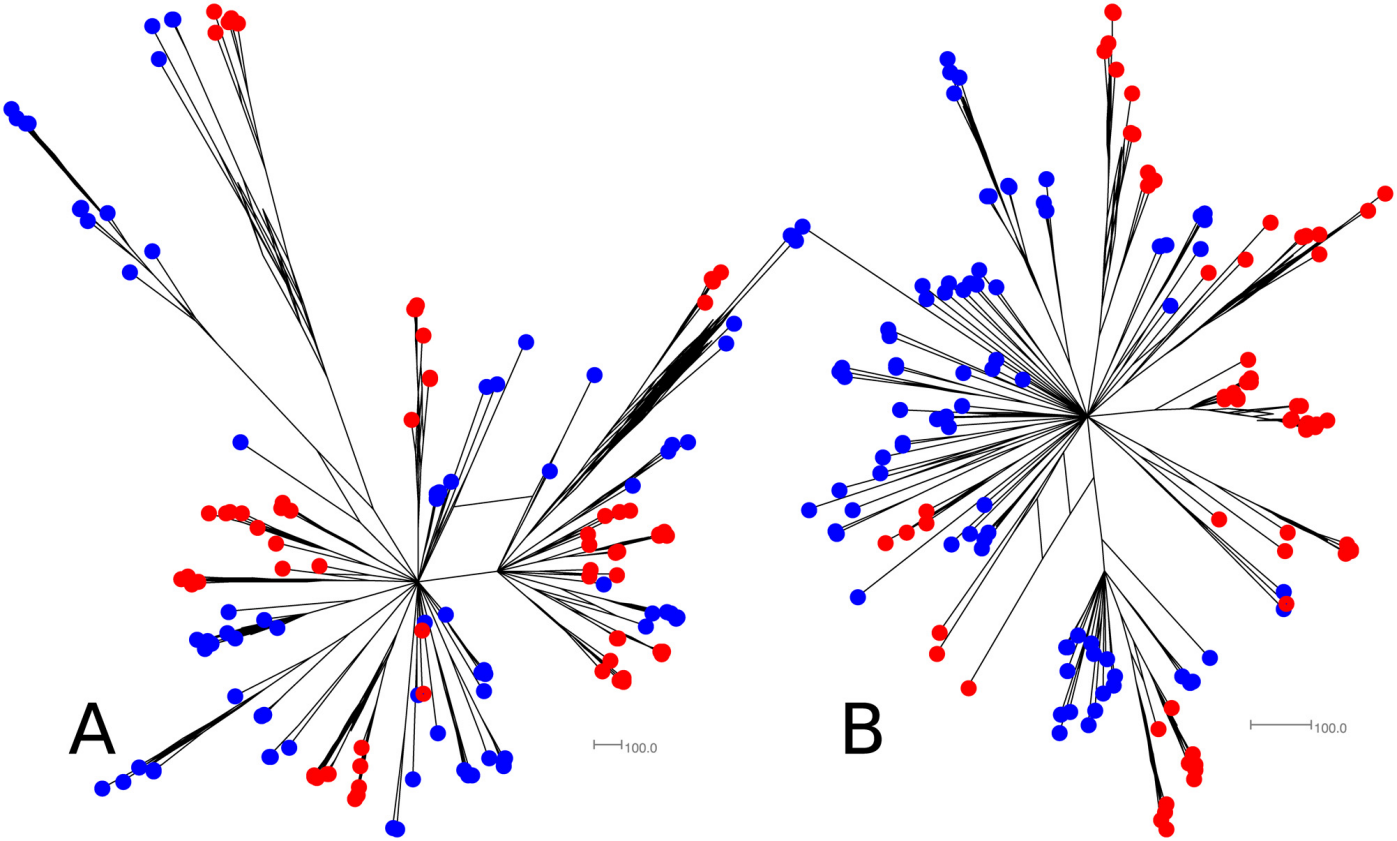
Consensus networks based on the posterior sets of trees for PI family 2. E2 (A) and NS5B (B) sequence alignments were evaluated independently. F2-G1 tips are blue and F2-G2 tips are red.

**Fig 6:**
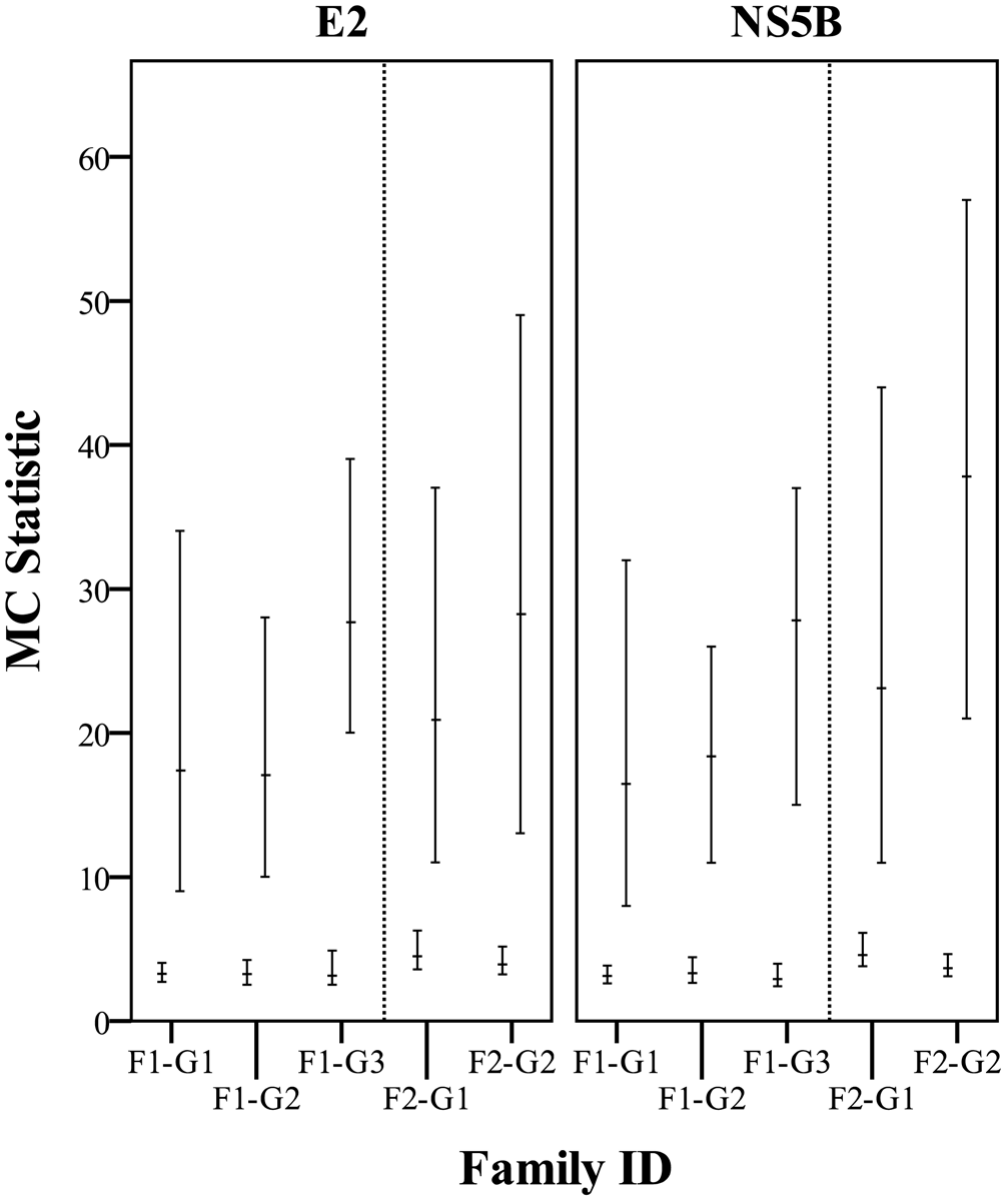
MC test and null distributions calculated with Befi-BaTS for each member of the PI families. Mean MC values are indicated by horizontal bars with the 95% confidence intervals of the null (lower bars) and test (upper bars) distributions indicated by the bars. All differences between null and test distributions are statistically significant as indicated by non-overlapping confidence intervals.

This sample set included two families of PI animals as indicated in Table 1. Family one consisted of three generations; F1-G1 was a 5 year old dam and the mother of F1-G2 who was 3 years old and in approximately the 4^th^ month of gestation with F1-G3. Family two consisted of two generations; F2-G1 was 3 years old and in approximately the 8^th^ month of gestation with F2-G2. Phylogenetic trees (S6 and S7 Figs) and consensus networks (Figs 4 and 5) were built as described previously using all clones obtained for each family. The association between clusters of tips on the posterior sets of trees (PSTs) and the generation of the PI family from which the respective sequences were derived was also evaluated using Befi-BaTS (see Fig 6 for maximum exclusive single-state clade size (MC) and S3 Table for all other Befi-BaTS output). A comparison of the means and 95% confidence intervals of the test and null distributions produced by Befi-BaTS shows that clonal sequences cluster by PI family generation on phylogenetic trees (Fig 6). These differences are significant as shown by the non-overlapping 95% confidence intervals.

Consensus networks depict star-like phylogenies with both distinct clustering based on family member as well as numerous interspersed sequences. As described previously, the E2 gene exhibits greater diversity than the NS5B gene.

### Tissue Compartmentalization

**Fig 7:**
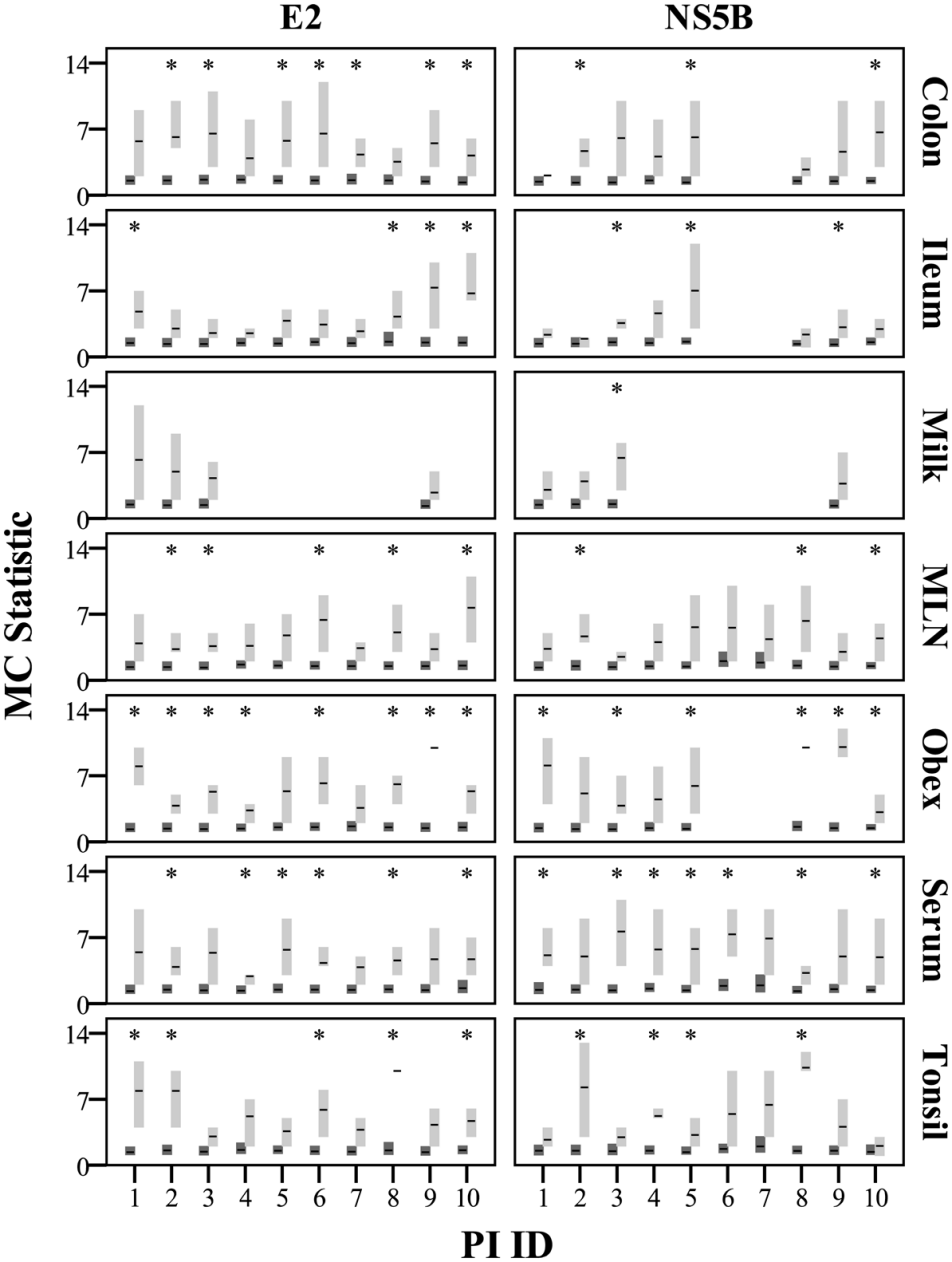
MC test and null distributions calculated with Befi-BaTS for each tissue compartment of each PI animal. Mean MC values are indicated by horizontal bars with the 95% confidence intervals of the null (dark grey) and test (light grey) distributions indicated by the vertical bars. Cases where the test and null distributions are significantly different and do not overlap have been marked with *.

The trait “tissue compartment” almost always clusters in the PSTs significantly more than expected by chance alone based on the Befi-BaTS/MC measurements (see Fig 7 for MC and S4 Table for all other Befi-BaTS output). Mean MC values were always higher for the test distributions than the null distributions and in many cases the 95% confidence intervals did not overlap. There appears to be no obvious trend in terms of the strength of this clustering when comparing different tissue compartments or different PI animals. It should also be noted that this association was stronger for the E2 gene than NS5B, although this difference was almost always non-significant as indicated by overlapping 95% confidence intervals. In addition to the statistical analysis, visual inspection of phylogenies identified a number of unique, cluster-specific mutations (S1 Table for family-specific mutations and S5 Table for tissue-specific mutations).

## Discussion

The sequence variability of cloned E2 and NS5B genomic regions was assessed to describe the level of viral diversity within and between PI cattle and, furthermore, to evaluate the ability of different body compartments to generate genetically diverse variants. Analysis of clones derived from the various tissue compartments revealed that only a small proportion of the variants were identical to the consensus sequence while the majority existed as a spectrum of mutants surrounding the consensus, supportive of a quasispecies distribution [27]. Patterns of intrahost viral variant distribution differed between all 10 PI cattle, with the N-terminal E2 ectodomain-encoding region being the most diverse (Fig 1). Although this finding is not novel, it does help to explain some of the differences observed in other analyses when comparing the E2 and NS5B genes. The NS5B gene on the other hand is less tolerant of amino acid changes due to its essential role in virus replication [28] and primarily synonymous changes were detected in a low proportion of the viruses (S1 Table). The E2 was demonstrated to be the more diverse region of the two, similar to other reports on *Pestiviral* [29, 30] and *Hepaciviral* genomes [31]. Earlier reports demonstrated sequence variation in the E2 gene with up to 15 amino acid residue differences in the clones analysed over an 11 month period [19]. However, due to the lack of an adaptive immune response and tolerance of the innate immune system induced by the BVDV proteins Npro and Erns [32], it can be postulated that virus selection in PI cattle is primarily influenced by tissue tropism requirements and other factors in the cellular environment. Of course, selective pressures on viral populations are multifactorial and other factors that were not controlled for, such as host diet and age for example, likely play important roles as well. Further research will be needed to understand these complex relationships.

We found statistically non-random associations between branching patterns and tissue compartments in the PSTs in almost all cases (Fig 7). Although the phylogenetic trees and consensus networks indicate tissue compartment clustering of isolates, the relationship is clearly complex and needs to be explained with viral and host biology in mind. Clusters with poor posterior probabilities should not be disregarded since non-tree like evolutionary events could cause poor support and biologically relevant polymorphisms such as single amino acid changes can be sufficient to significantly alter viral tropism or fidelity. For example, while the mechanism of compartmentalization of diverse variants within the obex has yet to be defined, several explanations that may not be reflected in phylogenetic inference could account for this observation. The central nervous system (CNS) represents an immunologically privileged site with limited immune reactivity due to the immune-modulating function of the neurovascular unit [33]. Therefore, it is possible that CNS-circulating virus does not have to maintain strict antigenic homology to the persisting viral strain [19, 34, 35] due to the lack of adaptive immune surveillance. In conjunction with this hypothesis, another major driver could be the selective expression of the cellular BVDV receptor CD46 [36], a regulator of complement activation that is present on all nucleated cells. It has been reported that CD46 is abundant at the blood-brain-barrier [37]. Cellular receptor density has been implicated in the production of larger viral populations and subsequent increases in pathogenicity, as shown with Coxsackie B virus in mice [38, 39]. In turn, more substantial quasispecies populations facilitate increasingly complex interactions, thus altering selective gains and variant survival potential [11].

Despite the indications for the CNS as an important viral reservoir, the clinical relevance has yet to be defined as it is unclear whether or not these variants will affect the course of disease or if they are able to circulate outside of the CNS. None of the PI animals examined expressed any CNS related disease signs. However, neurovirulent strains of BVDV have been reported and are associated with ataxia, recumbency, seizures, and CNS hypomyelination in calves [40, 41].

Genetic variability of BVDV within PI cattle was additionally assessed by phylogenetic analysis of two families of PI cattle. Within both families, the viral variability of E2 and NS5B sequences derived from the PI fetus (PI 4/F1-G3 and PI 10/F2-G2) was significantly reduced compared to the PI dam (PI 3/F1-G2 and PI 9/F2-G1 respectively) as shown in Fig 1. This is supportive of a transmission bottleneck, a phenomenon frequently observed in a variety of vertically transmitted viruses [42, 43]. The clustering patterns and accumulation of mutations we observed in the fetal E2 sequences also lead us to speculate that, analogous to HCV [44], evolution of BVDV following a transmission bottleneck can be at least partially defined by this envelope protein. The mechanism and severity of this bottleneck could not be established as we had access to only three such transmissions. In general, naturally occurring virus bottlenecks are poorly understood [45], but this bottleneck may be the result of a founder effect whereby an infection was established by one or a few variants, thus resulting in a vast reduction of genetic variability [46]. This is a likely scenario as the star-like consensus networks indicate that most isolates radiate from a small group of founder isolates (S4 and S5 Figs). Following this primary infection, viral particles quickly infect different tissue compartments and begin to diversify under the influence of tissue-specific factors. Furthermore, new infections could take place as gestation proceeds thereby increasing the variability. Repeated re-infections during 9 months of pregnancy could contribute to the establishment of a wide variety of maternal cow-specific variants before the calf is born. The comparison between PI 4 and PI 10 does not exclude this possibility as the 4 month old fetus had reduced variability while the viruses in the 8 month old fetus showed relatively limited reduction in variability compared to the adult, maternal cows. Another possible explanation is a selective sweep, which may account for the observed genetic restriction. In this case many variants are capable of establishment in the fetus, however relative fitness drives selective outgrowth of positively selected variants [47]. No strong indications of a fetus specific virus population were identified, therefore requiring further studies to test this hypothesis.

As opposed to the structural E2, NS5B is a non-structural gene encoding the RdRP. The absence of strong cluster formation in fetal F1-G3 indicates that there were no dominant subpopulations preferentially transmitted or selected within the host, suggesting that the NS5B is not a significant determinant of establishment of infection. Although many F1-G3 variants were interspersed, it is worth noting that 39% harboured the mutation D3779E located in the palm region of the polymerase, the location of the enzyme’s catalytic site [48]. It could therefore have an impact on enzyme function and viral phenotype.

Persistently infected cattle are the most significant propagators of BVDV and the identification of quasispecies in these hosts exemplifies the role of PI cattle in the generation of BVDV diversity. This diversity is essential for the virus to overcome bottlenecks and likely plays an important role in viral pathogenesis [49]. Synthesis of novel BVDV genetic diversity by PI cattle ensures continuous dispersal of new variants in the cattle population. Consequently, this will lead to unpredictable consequences for vaccine efficacy and these new BVDV variants may have novel epidemiological characteristics and altered virulence from currently circulating strains.

## Materials and Methods

### Ethics Statement

All procedures were approved by the University of Calgary animal care committee under protocol number VSACC SHC10R-16. This protocol adheres to the Canadian Council on Animal Care guidelines. All sampling was performed after obtaining permission from animal owners.

### Study Population and Sample Collection

Based on farmer or veterinarian clinical suspicion, BVDV PI animals were identified on farms by a combination of bulk milk PCR and serum antibody detection in non-vaccinated dairy herds (see description below). To identify individual PI cattle in antibody or PCR positive herds, serum samples were collected from the entire herd. From animals older than 6 months of age serum was collected whereas both serum and EDTA blood samples were obtained for animals younger than 6 months. Animals that were PCR positive for BVDV were retested three weeks later to confirm PI. Upon the owner’s preference a confirmatory serum sample was sent to Prairie Diagnostic Services (PDS, Saskatoon SK, Canada) or an ear notch was evaluated on farm using the SNAP BVDV Antigen test (IDEXX Laboratories, Inc. Westbrook ME, USA) according to the manufacturer's protocol. See Table 1 for description of farm and PI identification numbers. The final group of animals were all Holsteins from dairy farms in southern Alberta or Saskatchewan, Canada. All PI animals were euthanized and colon, ileum, milk, MLN, obex, serum, and tonsil samples were collected as part of a larger sampling process. All samples were stored overnight at 4°C, aliquoted the following day and stored at -80°C until further processing. Cross contamination of samples was avoided by discarding the exterior of all tissue samples when aliquots were prepared.

As a more systematic sampling approach was not possible due to the relative rarity of PI animals, an opportunistic sampling approach was used. This method of sampling was used over an infection trial primarily to better assess natural infection conditions (infectious dose, source of exposure, etc.), thus making the findings more relatable to naturally occurring PI.

### Antibody Detection

Serum samples from ten random heifers were subject to antibody detection using the HerdChek BVDV Antibody Test Kit (IDEXX Switzerland AG, Bern, Switzerland) following instructions of the manufacturer to determine if BVDV was present on the non-vaccinating farm.

### PCR and BVDV RNA Detection

The E.Z.N.A. Viral RNA Kit (Omega Bio-Tek, Norcross, GA, USA) was used to extract RNA from bulk milk according to the manufacturer’s instructions. PCR amplification of the 5’UTR region of the BVDV genome was carried out using the BluePrint One-Step RT-PCR kit (TAKARA Bio Inc., Otsu, Shiga, Japan) and primers 5’UTR-for and 5’UTR-rev as described by Ridpath et al. [50]. A 25μl reaction mixture was prepared consisting of 12.5μl of 2X One Step BluePrint Buffer, 10μl RNase free water, 1μl One Step BluePrint RT Enzyme Mix, a final concentration of each primer was 0.3μM in the reaction, and 0.5μl template RNA. PCR cycling conditions were as follows: 50°C for 30 minutes, 94°C for 2 minutes, 35 rounds of 98°C for 10 seconds, 52°C for 30 seconds, and 72°C for 1 minute, followed by a final extension of 72°C for 10 minutes.

Peripheral blood leucocytes (PBLs) were isolated from the EDTA blood samples by density gradient centrifugation (1.077 g/l, Lymfoprep Axis-Shield PoC AS, Oslo, Norway). Total RNA was extracted from PBLs and serum using the Mag-Bind Viral DNA/RNA Kit (Omega Bio-Tek, Norcross GA, USA) on the MagMAX Express-96 Deep Well Magnetic Particle Processor (Applied Biosystems, Burlington ON, Canada). Detection of BVDV RNA was performed using the real-time PCR VetMAX-Gold BVDV Detection Kit (Applied Biosystems, Burlington ON, Canada). All protocols were performed according to the manufacturer’s instructions on the CFX96 real time PCR system (Bio-Rad, Mississauga, ON, Canada).

### Amplification, Cloning and Sequencing of E2 and NS5b Gene Fragments

Viral RNA was extracted from tissue and excretion samples (colon, ileum, milk, MLN, obex, serum, and tonsil) from PI animals using the E.Z.N.A. Viral RNA Kit (Omega Bio-Tek, Norcross GA, USA) according to the manufacturer’s instructions. Prior to RNA extraction, tissue samples of approximately 1cm^3^ were soaked in 200μl PBS for 30 minutes and then homogenized using a pestle in a 1.5ml centrifuge tube. Tubes were vortexed, centrifuged and the supernatant was removed and used for subsequent extraction steps. Total RNA was used as template for PCR amplification of the E2 and NS5B regions of the genome using primers described previously [51, 52]. The BluePrint One-Step RT-PCR kit (TAKARA Bio Inc., Otsu, Shiga, Japan) was used and all incubations were performed in a T100 Thermal Cycler (Bio-Rad, Mississauga, ON, Canada). Cycling conditions for the E2 fragment were 50°C for 30 minutes, 94°C for 2 minutes, 35 rounds of 94°C for 30 seconds, 56°C for 1 minute, and 72°C for 1 minute, followed by a final extension of 72°C for 10 minutes. Cycling conditions for the NS5B fragment were 50°C for 30 minutes, 94°C for 2 minutes, 35 rounds of 94°C for 20 seconds, 50.5°C for 30 seconds, and 72°C for 30 seconds, and a final extension of 72°C for 15 minutes. Amplified PCR fragments were run on 1.5% agarose gels and bands of expected size (606bp for E2 and 1162bp for NS5B) were isolated and purified using the E.Z.N.A. Gel Extraction Kit (Omega Bio-Tek, Norcross, GA, USA) and then ligated into pGEM-T Easy vectors according to the manufacturer’s instructions (Promega, Madison WI, USA). Ampicillin resistant recombinant colonies were selected via a blue/white screening. Plasmids containing inserts were purified from bacteria using the E.Z.N.A. Plasmid Mini Kit (Omega Bio-Tek, Norcross GA, USA) and a minimum of 10 clones per tissue were sequenced on an automated ABI sequencer (Applied Biosystems, Streetsville ON, Canada) at Eurofins MWG Operon (Huntsville AL, USA) using T7 and SP6 primers.

Regarding the fidelity of the DNA polymerase used, the expected number of polymerase-induced errors is far less than what would be required to influence our findings. Assuming an error rate of 1 in every 15750 nucleotides (or 4.5X less than the 1 in 3500 of *Taq* DNA polymerase) and a random error distribution, we would expect 63 polymerase-induced errors in these data resulting in less than 0.05 errors per sequence analyzed. As such, polymerase-induced errors could not have altered the sequence data sufficiently to influence the conclusions reached in this study. Furthermore, any polymerase-induced errors would act only to weaken the observed patterns. The use of a higher fidelity enzyme would therefore only strengthen these findings, not refute them.

### Alignment and Phylogenetic Analysis

Vectors and primers were trimmed prior to assembly of the sequence contigs using Geneious vR8 [53]. Nucleotide alignments were constructed using multiple sequence comparison by log-expectation (MUSCLE) [54]. The overall genetic diversity within each animal was measured on the aligned sequences using MEGA 5 [55]. The Tajima-Nei model [56] was used to calculate corrected nucleotide distances.

Model testing was performed using Topali v2.5 with MrBayes tree generation for each alignment [57, 58]. The Bayesian information criterion was used for model selection. Sequence alignments were processed using BEAUti v1.8.1 using the appropriate substitution models to produce input files for BEAST v1.8.1 [59]. Proper priors were specified using prior knowledge of BVDV mutation rates [60] with other priors being left uninformed. Analyses were run for 50 million steps and a 10% burnin was used to ensure convergence and proper sampling of all traces. Tracer v1.6 was used to ensure the quality of all runs prior to further analysis. Tree files were then processed with TreeAnnotator v1.8.1 to produce a single tree using median heights and a 10% burn-in that could be visualized in FigTree v1.4.2.

Given the rapid evolution of BVDV and that PI production may result from *in utero* infection with a small number of founder viral particles, phylogenetic networks were built to better accommodate potentially non-tree like evolutionary events. Consensus networks [61] were produced using SplitsTree v4.13.1 from the original tree files (down sampled 1:10 with a 10% burnin and a split inclusion cutoff of 25%) [62]. Tree files with a 10% burnin were also analyzed using Befi-BaTS to test if tissue compartments or host of origin clustered together on the phylogenies more than would be expected by chance alone [63]. Several statistics were evaluated to determine if the trait distribution was non-random on the trees (association index (AI) [64], parsimony score (PS) [65], unique fractions (UniFrac) [66], nearest taxa index (NTI) [67], nearest relatedness index (NRI) [68], and phylogenetic diversity (PD) [69]). The maximum monophyletic clade (MC) was also calculated for each tissue compartment in each animal [63]. 100 replicates were used for these calculations as recommended by the developer.

## Supporting Information

S1 Fig Phylogenetic tree of E2 gene fragment consensus sequences for each PI animal. Tips are coloured according to the farm of origin with posterior probabilities noted at their respective nodes. Several reference sequences are indicated with their GenBank accession numbers.
(TIF)

S2 Fig Phylogenetic trees based on E2 gene fragment alignments. The PI ID is indicated to the bottom right of each tree. Branches are shaded according to their posterior probability (0 = orange, 1 = black) and the tip labels are coloured according to their tissue of origin (tonsil = yellow, colon = green, MLN = blue, obex = purple, serum = orange, ileum = teal, milk = red). The long branches in the tree for PI1 are the result of incongruous phylogenetic signals in the data which produce negative median branch lengths. Note: trees are not all drawn to the same scale.
(TIF)

S3 Fig Phylogenetic trees based on NS5B gene fragment alignments. The PI ID is indicated to the bottom right of each tree. Branches are shaded according to their posterior probability (0 = orange, 1 = black) and the tip labels are coloured according to their tissue of origin (tonsil = yellow, colon = green, MLN = blue, obex = purple, serum = orange, ileum = teal, milk = red). Note: trees are not all drawn to the same scale.
(TIF)

S4 Fig Consensus networks based on the posterior tree sets used to construct the trees in S2 Fig. Tips are coloured according to their tissue of origin (tonsil = yellow, colon = green, MLN = blue, obex = purple, serum = orange, ileum = teal, milk = red). The PI ID is indicated next to each network.
(TIF)

S5 Fig Consensus networks based on the posterior tree sets used to construct the trees in S3 Fig. Tips are coloured according to their tissue of origin (tonsil = yellow, colon = green, MLN = blue, obex = purple, serum = orange, ileum = teal, milk = red). The PI ID is indicated next to each network.
(TIF)

S6 Fig Phylogenetic trees based on E2 gene fragment alignment at the family level. Family 1 is shown in tree (A) and family 2 in tree (B). Branches are shaded according to their posterior probability (0 = orange, 1 = black) and the tip labels are coloured according to their generation within the PI family. Note: trees are not all drawn to the same scale.
(TIF)

S7 Fig Phylogenetic trees based on NS5B gene fragment alignment at the family level. Family 1 is shown in tree (A) and family 2 in tree (B). Branches are shaded according to their posterior probability (0 = orange, 1 = black) and the tip labels are coloured according to their generation within the PI family. Note: trees are not all drawn to the same scale.
(TIF)

S1 Table Descriptions of clustered E2 and NS5B mutations in two families of PI cattle. The letter in the cluster name identifies the cluster and the subsequent number indicates clusters that are nested. For example, cluster K.1 is nested within cluster K. All mutations are denoted by the original nucleotide/amino acid, the genome position, and the new nucleotide/amino acid. All genome positions are relative to the NADL (GenBank# M31182) to allow for consistent numbering. Underlined mutations indicate mutations that were observed only in the fetus.
(DOCX)

S2 Table Intrahost diversity of E2 and NS5B sequence alignments. The mean diversity and standard error of the mean (SEM) statistics of intrahost E2 and NS5B sequence alignments as shown in Fig 1.
(DOCX)

S3 Table Befi-BaTS analysis of posterior sets of trees derived from gene alignments for each PI family. Tree size and internal branch size are average measures of all trees in the set while the remaining parameters are measures of the association between family generation and phylogenetic clustering. All measures of clustering have non-overlapping 95% confidence intervals relative to null distributions with the exception of UniFrac intervals which do overlap in all cases.
(DOCX)

S4 Table Befi-BaTS analysis of posterior sets of trees derived from gene alignments for each PI animal. Tree size and internal branch size are average measures of all trees in the set while the remaining parameters are measures of the association between tissue compartment and phylogenetic clustering. All measures of clustering have non-overlapping 95% confidence intervals relative to null distributions with the exception of UniFrac intervals, which do overlap in all cases.
(DOCX)

S5 Table Position and frequency of E2 mutations in tissue-specific clusters. The first number of the cluster name indicates the PI in which the cluster was identified. Compartmentalized variants were found in tissues of the obex and/or tonsil in four of ten PI cattle in this study. No mutations were shared among multiple PI hosts from different farms, although both PI 1 and 9 had mutations at position 170 in obex clusters (underlined). All mutations are denoted by the original nucleotide/amino acid, the genome position, and the new nucleotide/amino acid. All genome positions are relative to the NADL (GenBank# M31182) to allow for consistent numbering.
(DOCX)
