# Supplementary figures and images for: Genetic Variability of Bovine Viral Diarrhea Virus and Evidence for a Possible Genetic Bottleneck during Vertical Transmission in Persistently Infected Cattle
Source: PLoS One. 2015 Jul 1;10(7):e0131972. doi: 10.1371/journal.pone.0131972 (PMC4488595; doi:10.1371/journal.pone.0131972)

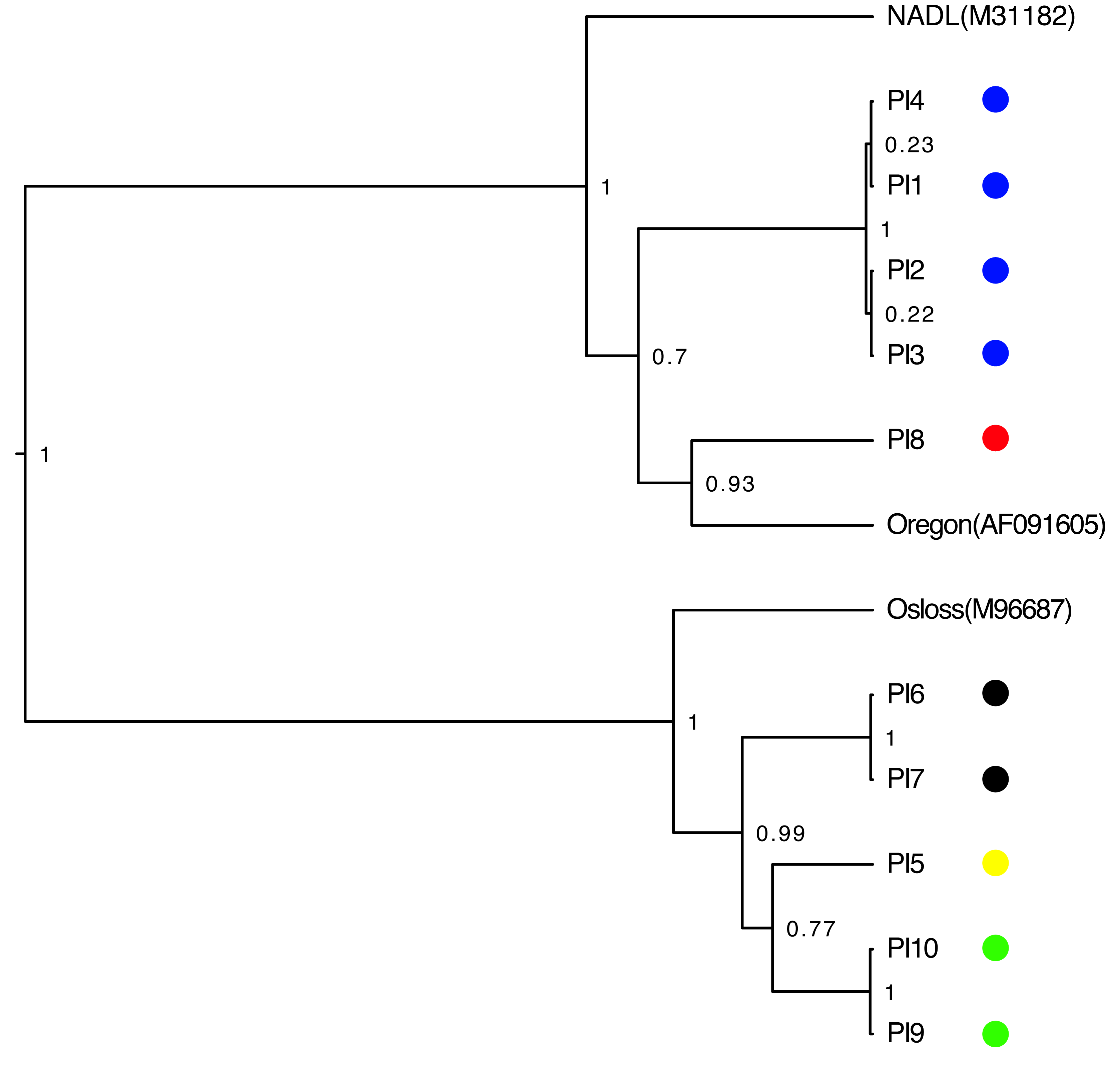

Supplement: S1 Fig — Tips are coloured according to the farm of origin with posterior probabilities noted at their respective nodes. Several reference sequences are indicated with their GenBank accession numbers. (TIF) [file pone.0131972.s001.tif]

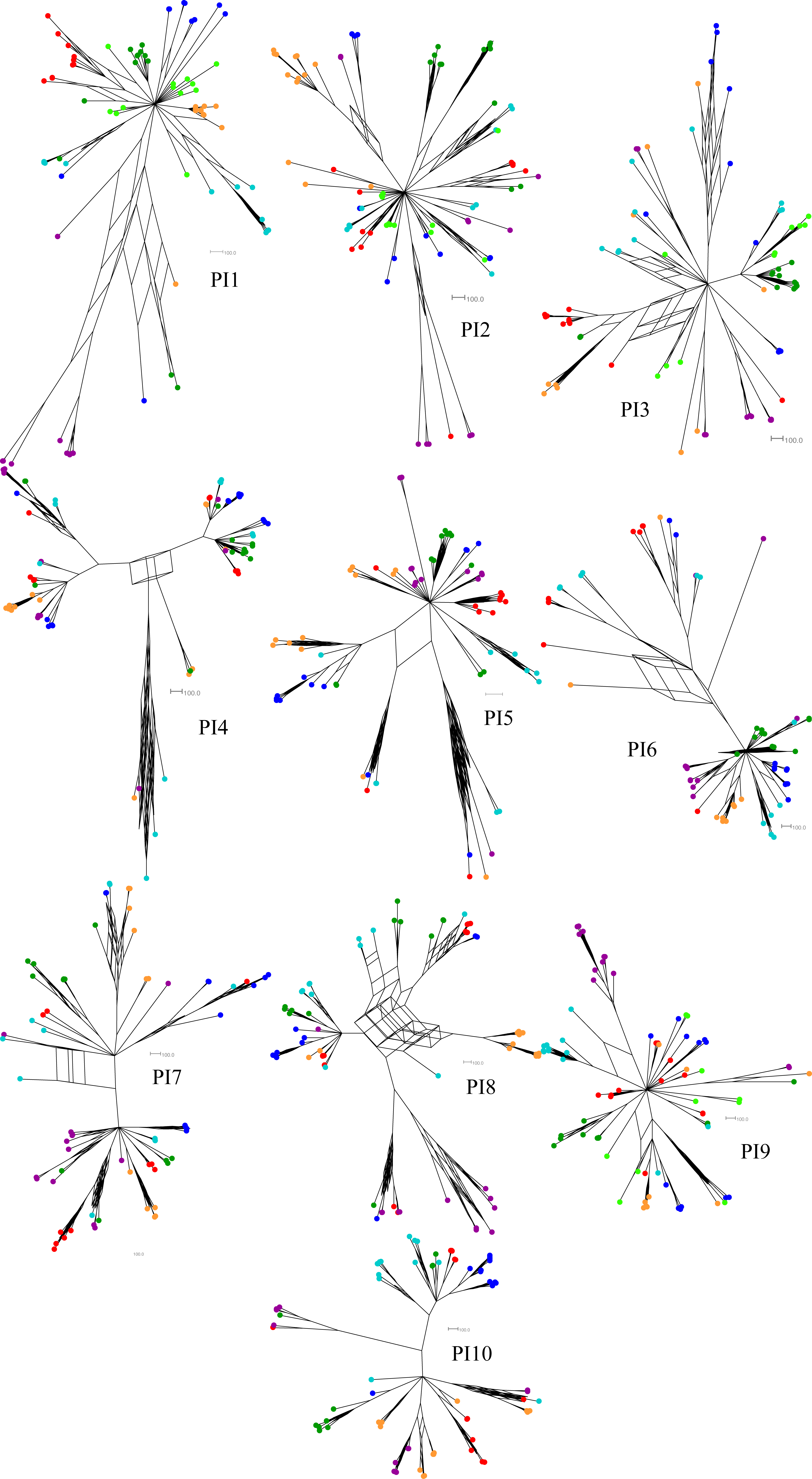

Supplement: S4 Fig — Tips are coloured according to their tissue of origin (tonsil = yellow, colon = green, MLN = blue, obex = purple, serum = orange, ileum = teal, milk = red). The PI ID is indicated next to each network. (TIF) [file pone.0131972.s004.tif]

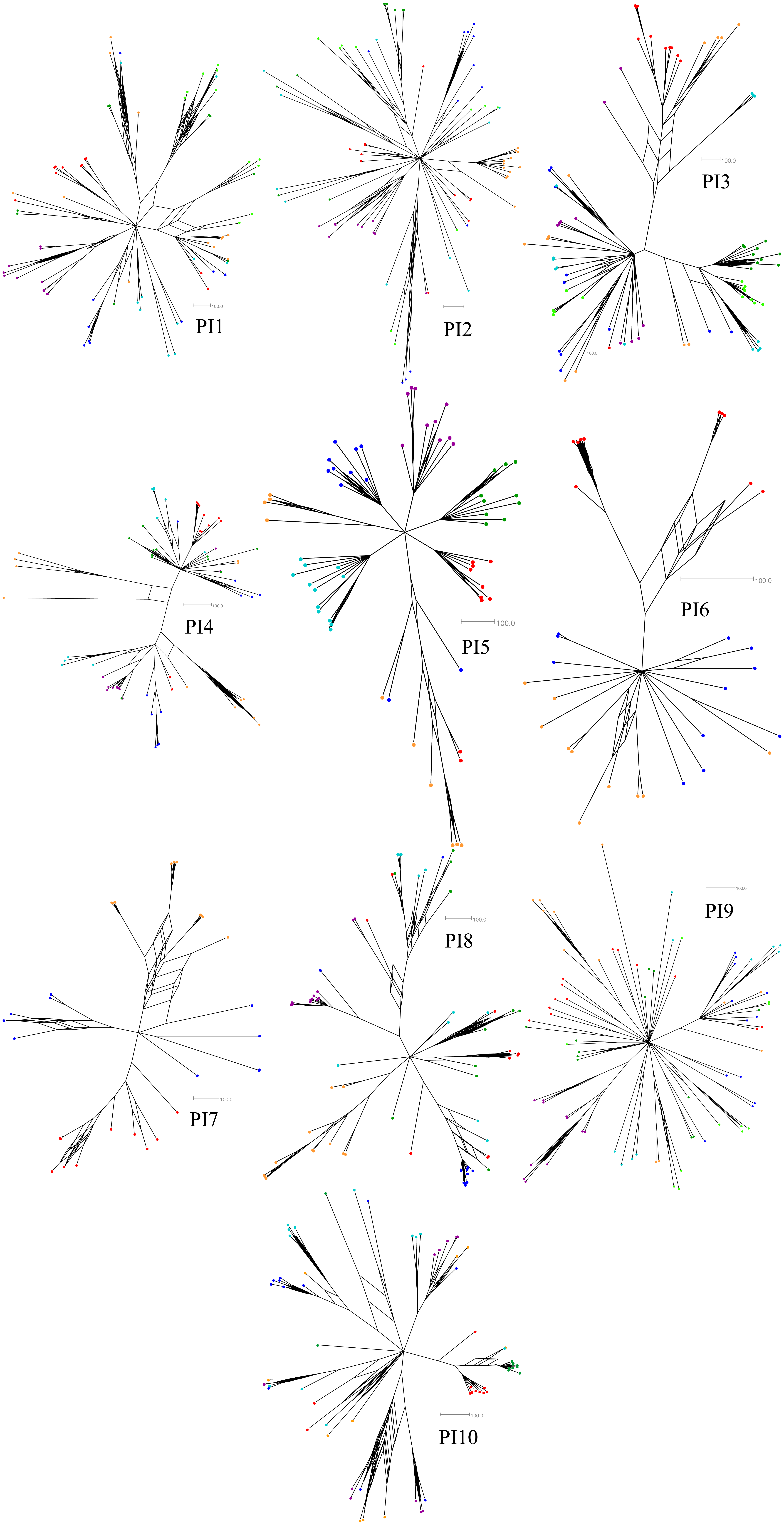

Supplement: S5 Fig — Tips are coloured according to their tissue of origin (tonsil = yellow, colon = green, MLN = blue, obex = purple, serum = orange, ileum = teal, milk = red). The PI ID is indicated next to each network. (TIF) [file pone.0131972.s005.tif]

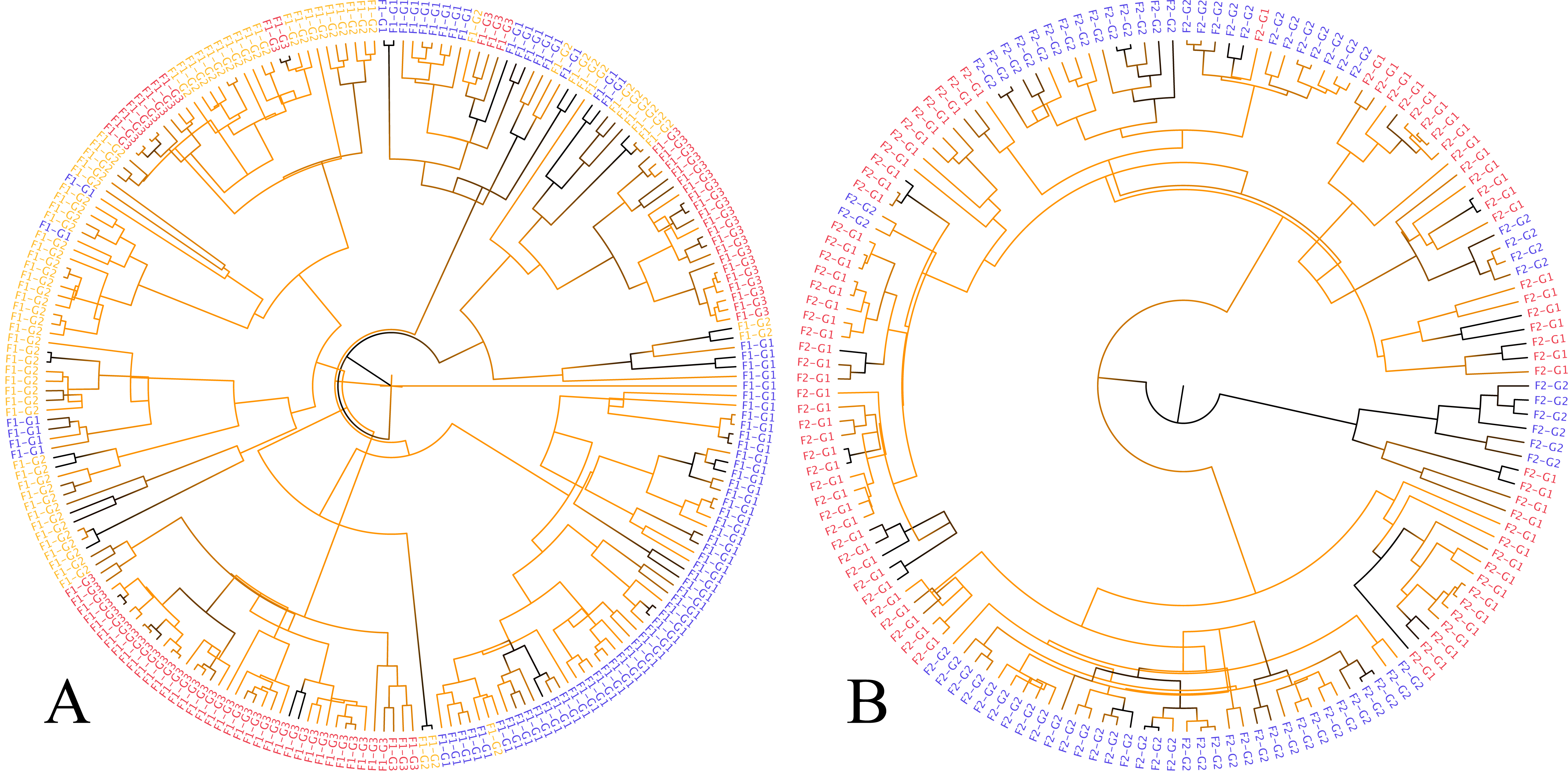

Supplement: S6 Fig — Family 1 is shown in tree (A) and family 2 in tree (B). Branches are shaded according to their posterior probability (0 = orange, 1 = black) and the tip labels are coloured according to their generation within the PI family. Note: trees are not all drawn to the same scale. (TIF) [file pone.0131972.s006.tif]

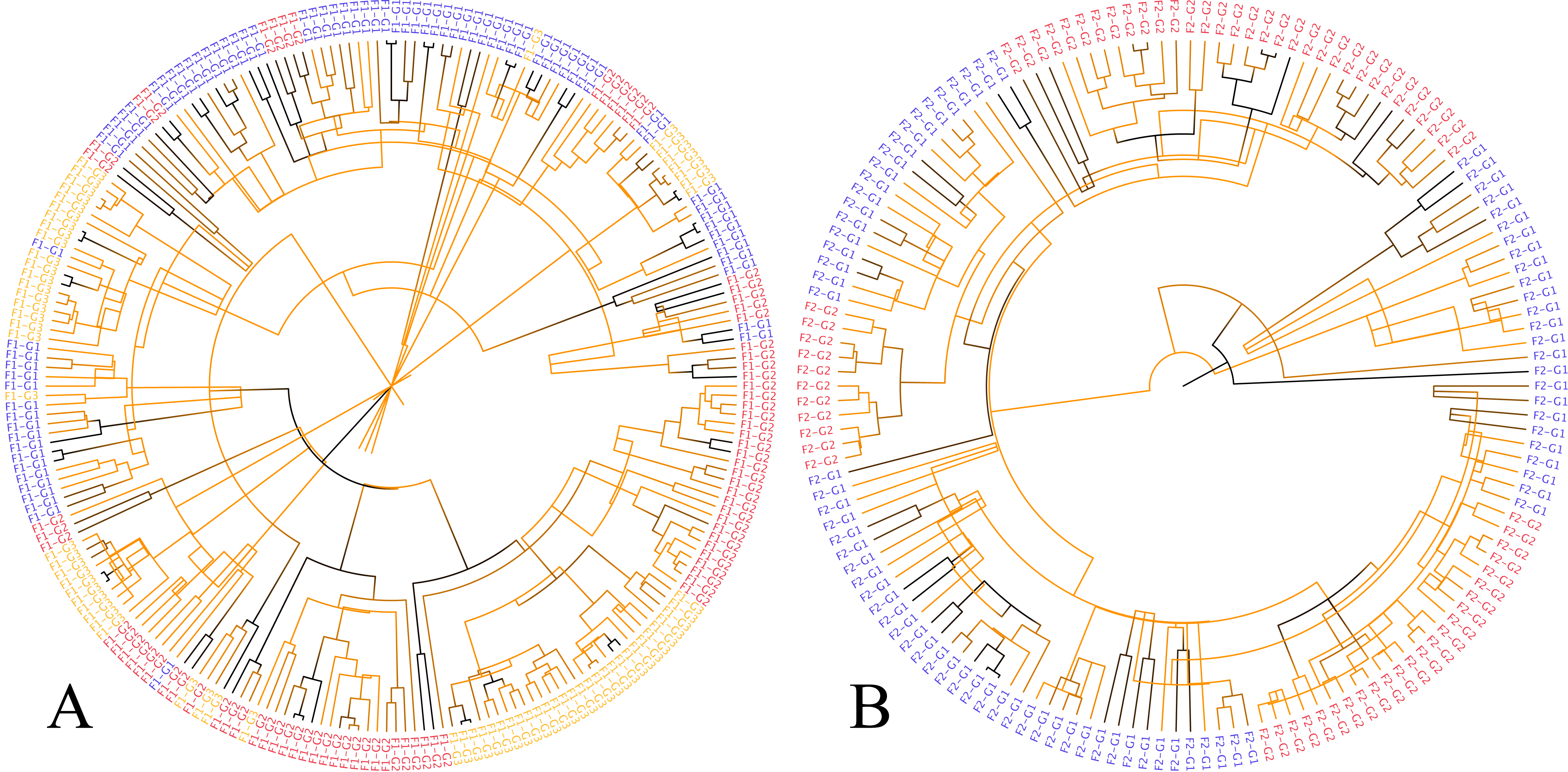

Supplement: S7 Fig — Family 1 is shown in tree (A) and family 2 in tree (B). Branches are shaded according to their posterior probability (0 = orange, 1 = black) and the tip labels are coloured according to their generation within the PI family. Note: trees are not all drawn to the same scale. (TIF) [file pone.0131972.s007.tif]
