# Supplementary material for: Genetic Variability of Bovine Viral Diarrhea Virus and Evidence for a Possible Genetic Bottleneck during Vertical Transmission in Persistently Infected Cattle
Source: PLoS One. 2015 Jul 1;10(7):e0131972. doi: 10.1371/journal.pone.0131972 (PMC4488595; doi:10.1371/journal.pone.0131972)
Supplement: S1 Table — The letter in the cluster name identifies the cluster and the subsequent number indicates clusters that are nested. For example, cluster K.1 is nested within cluster K. All mutations are denoted by the original nucleotide/amino acid, the genome position, and the new nucleotide/amino acid. All genome positions are relative to the NADL (GenBank# M31182) to allow for consistent numbering. Underlined mutations indicate mutations that were observed only in the fetus. (DOCX) [file pone.0131972.s008.docx]

Table S1: Descriptions of clustered E2 and NS5B mutations in two families of PI cattle.

|  | **Mutation** | **Amino Acid change** |
| --- | --- | --- |
| **E2** | | |
| **Family 1** |  |  |
| Cluster A | C2827T | syn |
| Cluster A.1 | C2827T; A2670G | syn; H762R |
| Cluster B | G2698A | syn |
| Cluster B.1 | G2698A; C2520T | syn; P712L |
| **Family 2** |  |  |
| Cluster C | C2541T; G2548T; C2627T | T719I; R721H ; syn |
| Cluster D | C2627T | syn |
| Cluster E | T2682C; A2806G | L766S; K807N |
| Cluster F | A2803G | syn |
| **NS5B** | | |
| **Family 1** |  |  |
| Cluster G | G10477A; C11005T; G11422A | syn |
| Cluster H | C11338A | D3779E |
| **Family 2** |  |  |
| Cluster I | T11494C | syn |
| Cluster I.1 | T11494C; A11335T | syn |
| Cluster I.2 | T11494C; A11335T; A11380G | syn |
| Cluster J | G11482A; C11521T | syn |
| Cluster K | C11299T | syn |
| Cluster K.1 | C11299T; C10657T | syn |

Syn=synonymous mutation

The letter in the cluster name identifies the cluster and the subsequent number indicates clusters that are nested. For example, cluster K.1 is nested within cluster K. All mutations are denoted by the original nucleotide/amino acid, the genome position, and the new nucleotide/amino acid. All genome positions are relative to the NADL (GenBank# M31182) to allow for consistent numbering. Underlined mutations indicate mutations that were observed only in the fetus.
