# Supplementary material for: Genetic Variability of Bovine Viral Diarrhea Virus and Evidence for a Possible Genetic Bottleneck during Vertical Transmission in Persistently Infected Cattle
Source: PLoS One. 2015 Jul 1;10(7):e0131972. doi: 10.1371/journal.pone.0131972 (PMC4488595; doi:10.1371/journal.pone.0131972)
Supplement: S2 Table — The mean diversity and standard error of the mean (SEM) statistics of intrahost E2 and NS5B sequence alignments as shown in Fig 1. (DOCX) [file pone.0131972.s009.docx]

Table S2: Intrahost diversity of E2 and NS5B sequence alignments

|  | **E2** | | **NS5B** | |
| --- | --- | --- | --- | --- |
| **PI ID** | **Mean** | **SEM** | **Mean** | **SEM** |
| 1 | 0.01051 | 0.000140 | 0.00660 | 0.000053 |
| 2 | 0.01106 | 0.000119 | 0.00664 | 0.000049 |
| 3 | 0.01229 | 0.000118 | 0.00716 | 0.000058 |
| 4 | 0.00909 | 0.000105 | 0.00378 | 0.000044 |
| 5 | 0.00720 | 0.000100 | 0.00366 | 0.000044 |
| 6 | 0.00931 | 0.000116 | 0.00340 | 0.000055 |
| 7 | 0.00952 | 0.000104 | 0.00432 | 0.000119 |
| 8 | 0.01230 | 0.000140 | 0.00554 | 0.000048 |
| 9 | 0.01147 | 0.000109 | 0.00555 | 0.000045 |
| 10 | 0.00988 | 0.000115 | 0.00497 | 0.000045 |

The mean diversity and standard error of the mean (SEM) statistics of intrahost E2 and NS5B sequence alignments as shown in Figure 2.
