# Supplementary material for: Genetic Variability of Bovine Viral Diarrhea Virus and Evidence for a Possible Genetic Bottleneck during Vertical Transmission in Persistently Infected Cattle
Source: PLoS One. 2015 Jul 1;10(7):e0131972. doi: 10.1371/journal.pone.0131972 (PMC4488595; doi:10.1371/journal.pone.0131972)
Supplement: S4 Table — Tree size and internal branch size are average measures of all trees in the set while the remaining parameters are measures of the association between tissue compartment and phylogenetic clustering. All measures of clustering have non-overlapping 95% confidence intervals relative to null distributions with the exception of UniFrac intervals, which do overlap in all cases. (DOCX) [file pone.0131972.s011.docx]

Table S4: Befi-BaTS analysis of posterior sets of trees derived from gene alignments for each PI animal

| **E2** | | | | | | | | | | |
| --- | --- | --- | --- | --- | --- | --- | --- | --- | --- | --- |
|  | **1** | **2** | **3** | **4** | **5** | **6** | **7** | **8** | **9** | **10** |
| **Tree size** | 35264.227 | 28398.643 | 30022.15 | 17638.662 | 17336.65 | 20946.404 | 24081.705 | 23461.344 | 31639.674 | 21479.693 |
| **Internal branch size** | 19096.357 | 12961.577 | 14764.472 | 8733.385 | 8435.764 | 9380.544 | 10918.816 | 12087.89 | 14688.507 | 11163.408 |
| **AI** | 1.016 | 1.909 | 1.878 | 2.508 | 1.639 | 1.507 | 1.95 | 1.297 | 1.953 | 1.349 |
| **PS** | 20.249 | 28.537 | 29.202 | 36.003 | 26.616 | 23.307 | 32.495 | 24.472 | 28.392 | 23.251 |
| **UniFrac** | 0.53 | 0.536 | 0.472 | 0.296 | 0.471 | 0.481 | 0.405 | 0.492 | 0.501 | 0.444 |
| **NTI** | 7364.182 | 6249.851 | 6807.196 | 5501.527 | 4649.351 | 5608.694 | 6565.035 | 5886.403 | 7342.671 | 5627.273 |
| **NRI** | 109422.555 | 88182.969 | 97891.945 | 109806.422 | 77349.773 | 79155.539 | 117992.664 | 120217 | 102580.961 | 105019.109 |
| **PD** | 56457.566 | 42068.457 | 47742.793 | 33953.895 | 28090.033 | 32343.119 | 39755.746 | 38764.039 | 47939.359 | 34654.926 |
| **NS5B** | | | | | | | | | | |
| **Tree size** | 19664.689 | 23581.922 | 26228.453 | 12353.822 | 17065.777 | 4257.953 | 6928.772 | 10681.806 | 26053.311 | 10131.686 |
| **Internal branch size** | 7655.047 | 9040.315 | 10381.921 | 5636.81 | 7759.997 | 1675.812 | 3842.336 | 4331.225 | 10192.117 | 3517.5 |
| **AI** | 2.433 | 2.328 | 1.676 | 1.723 | 0.918 | 0.375 | 0.203 | 1.562 | 1.812 | 2.479 |
| **PS** | 33.639 | 31.197 | 28.028 | 24.264 | 16.75 | 6.663 | 5.494 | 26.104 | 28.966 | 32.774 |
| **UniFrac** | 0.501 | 0.498 | 0.502 | 0.471 | 0.525 | 0.686 | 0.713 | 0.481 | 0.513 | 0.253 |
| **NTI** | 4804.978 | 5939.672 | 5766.131 | 3343.203 | 3898.921 | 1963.61 | 2346.716 | 2984.388 | 5880.546 | 3475.245 |
| **NRI** | 56682.891 | 68047.313 | 87969.539 | 55048.008 | 53429.055 | 21088.281 | 33799.738 | 39995.063 | 87538.484 | 39403.863 |
| **PD** | 28747.08 | 33693.68 | 37947.855 | 19320.279 | 24779.092 | 4942.274 | 8463.891 | 15832.44 | 38492.547 | 16388.541 |

Tree size and internal branch size are average measures of all trees in the set while the remaining parameters are measures of the association between tissue compartment and phylogenetic clustering. All measures of clustering have non-overlapping 95% confidence intervals relative to null distributions with the exception of UniFrac intervals, which do overlap in all cases.
