# Supplementary material for: Genetic Variability of Bovine Viral Diarrhea Virus and Evidence for a Possible Genetic Bottleneck during Vertical Transmission in Persistently Infected Cattle
Source: PLoS One. 2015 Jul 1;10(7):e0131972. doi: 10.1371/journal.pone.0131972 (PMC4488595; doi:10.1371/journal.pone.0131972)
Supplement: S5 Table — The first number of the cluster name indicates the PI in which the cluster was identified. Compartmentalized variants were found in tissues of the obex and/or tonsil in four of ten PI cattle in this study. No mutations were shared among multiple PI hosts from different farms, although both PI 1 and 9 had mutations at position 170 in obex clusters (underlined). All mutations are denoted by the original nucleotide/amino acid, the genome position, and the new nucleotide/amino acid. All genome positions are relative to the NADL (GenBank# M31182) to allow for consistent numbering. (DOCX) [file pone.0131972.s012.docx]

Table S5: Position and frequency of E2 mutations in tissue-specific clusters.

|  | **Compartment** | **Total # clones in cluster** | **Nucleotide position and change** | **Amino Acid**  **Change** |
| --- | --- | --- | --- | --- |
| **Cluster 1.1** | Obex | 6 | T2600C | W867R |
|  |  |  | C2631T | P877L |
|  |  |  | A2642G, A2644C | R881G |
|  |  |  | A2644C | R881S |
|  |  |  | A2723G | R908G |
|  |  |  | C2779T | Syn |
|  |  |  | A2805G | K935R |
| **Cluster 2.1** | Tonsil | 9 | T2600C | W867R |
| **Cluster 8.1** | Obex | 7 | T2595C | I865T |
|  |  |  | T2628C | F876S |
|  |  |  | G2702A | E901K |
| **Cluster 8.2** | Tonsil | 10 | T2520C | L840P |
|  |  |  | A2552G | K851E |
|  |  |  | G2686A | Syn |
|  |  |  | G2702A | E901K |
|  |  |  | T2770C | Syn |
| **Cluster 9.1** | Obex | 10 | C2589T | T863I |
|  |  |  | A2630G, T2631C | I877A |
|  |  |  | T2631C | I877T |

^*^Position on the NADL (GenBank# M31182) genome sequence

Syn= Synonymous mutation

The first number of the cluster name indicates the PI in which the cluster was identified. Compartmentalized variants were found in tissues of the obex and/or tonsil in four of ten PI cattle in this study. No mutations were shared among multiple PI hosts from different farms, although both PI 1 and 9 had mutations at position 170 in obex clusters (underlined). All mutations are denoted by the original nucleotide/amino acid, the genome position, and the new nucleotide/amino acid. All genome positions are relative to the NADL (GenBank# M31182) to allow for consistent numbering.
